# The implications of silent transmission for the control of COVID-19 outbreaks

**DOI:** 10.1073/pnas.2008373117

**Published:** 2020-07-06

**Authors:** Seyed M. Moghadas, Meagan C. Fitzpatrick, Pratha Sah, Abhishek Pandey, Affan Shoukat, Burton H. Singer, Alison P. Galvani

**Affiliations:** ^a^Agent-Based Modelling Laboratory, York University, Toronto, ON M3J 1P3, Canada;; ^b^Center for Infectious Disease Modeling and Analysis, Yale School of Public Health, New Haven, CT 06510;; ^c^Center for Vaccine Development and Global Health, University of Maryland School of Medicine, Baltimore, MD 21201;; ^d^Emerging Pathogens Institute, University of Florida, Gainesville, FL 32610

**Keywords:** COVID-19, contact tracing, case isolation

## Abstract

Since the emergence of coronavirus disease 2019 (COVID-19), unprecedented movement restrictions and social distancing measures have been implemented worldwide. The socioeconomic repercussions have fueled calls to lift these measures. In the absence of population-wide restrictions, isolation of infected individuals is key to curtailing transmission. However, the effectiveness of symptom-based isolation in preventing a resurgence depends on the extent of presymptomatic and asymptomatic transmission. We evaluate the contribution of presymptomatic and asymptomatic transmission based on recent individual-level data regarding infectiousness prior to symptom onset and the asymptomatic proportion among all infections. We found that the majority of incidences may be attributable to silent transmission from a combination of the presymptomatic stage and asymptomatic infections. Consequently, even if all symptomatic cases are isolated, a vast outbreak may nonetheless unfold. We further quantified the effect of isolating silent infections in addition to symptomatic cases, finding that over one-third of silent infections must be isolated to suppress a future outbreak below 1% of the population. Our results indicate that symptom-based isolation must be supplemented by rapid contact tracing and testing that identifies asymptomatic and presymptomatic cases, in order to safely lift current restrictions and minimize the risk of resurgence.

Many countries, including the United States, are struggling to control coronavirus disease 2019 (COVID-19) outbreaks. Understanding how silent infections that are in the presymptomatic phase or asymptomatic contribute to transmission will be fundamental to the success of postlockdown control strategies. The effectiveness of symptom-based interventions depends on the fraction of infections that are asymptomatic, the infectiousness of those asymptomatic cases, and the duration and infectiousness of the presymptomatic phase. Empirical studies have indicated that individuals may be most infectious during the presymptomatic phase (1), an unusual characteristic for a respiratory infection.

To quantify the population-level contribution of silent transmission to COVID-19 spread, we extended our previous model (2, 3) to include asymptomatic infections and the presymptomatic stage, parameterized with data regarding the trajectory of symptom onset and the proportion of secondary cases generated in each stage of infection (1, 4). As empirical studies indicate that asymptomatic infections account for 17.9 to 30.8% of all infections (5, 6), for both of these values, we quantified the proportion of the attack rate attributable to transmission during presymptomatic, asymptomatic, and symptomatic stages. Furthermore, this quantification was combined with a series of scenario analyses to identify the level of isolation required for symptomatic or silently infected individuals, to suppress the attack rate below 1%. Our results highlight the role of silent transmission as the primary driver of COVID-19 outbreaks and underscore the need for mitigation strategies, such as contact tracing, that detect and isolate infectious individuals prior to the onset of symptoms.

## Results

**Fig. 1.**
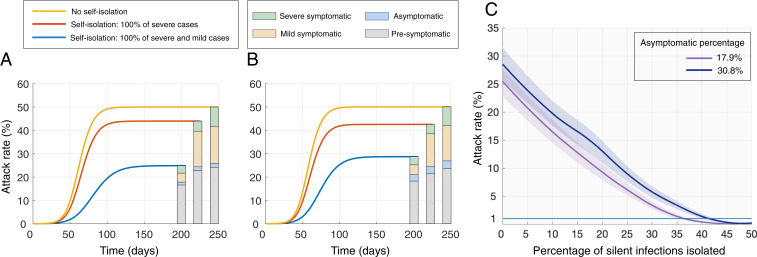
Attack rates when the proportion of infections that are asymptomatic is (*A*) 17.9% and (*B*) 30.8%, for scenarios of case isolation including none (yellow), all severe cases (red), and all symptomatic cases (blue). Bars indicate the proportion of attack rate attributable to transmission in different stages of infections. (*C*) Attack rate when a percentage of silent (i.e., presymptomatic and asymptomatic) infections are detected and isolated in addition to immediate isolation of both mild and severe symptomatic cases.

Translating clinical data on infectiousness and symptoms (1) to population-level epidemiological impact, our results indicate that the majority of transmission is attributable to people who are not exhibiting symptoms, either because they are still in the presymptomatic stage or the infection is asymptomatic (Fig. 1). Specifically, if 17.9% of infections are asymptomatic (5), we found that the presymptomatic stage and asymptomatic infections account for 48% and 3.4% of transmission, respectively (Fig. 1*A*). Considering a greater asymptomatic proportion of 30.8% reported in another empirical study (6), the presymptomatic phase and asymptomatic infections account for 47% and 6.6% of transmission, respectively (Fig. 1*B*). Consequently, even immediate isolation of all symptomatic cases is insufficient to achieve control (Fig. 1). Specifically, mean attack rates remain above 25% of the population when 17.9% of infections are asymptomatic and above 28% when 30.8% of infectious are asymptomatic.

Given the inadequacy of symptom-based isolation to control COVID-19 outbreaks, we considered the synergistic impact of isolation for presymptomatic and asymptomatic infections. Combined with case isolation, our results indicate that 33% and 42% detection and isolation of silent infections would be needed to suppress the attack rate below 1%, for asymptomatic proportions of 17.9% and 30.8%, respectively (Fig. 1*C*).

## Discussion

Our results indicate that silent disease transmission during the presymptomatic and asymptomatic stages are responsible for more than 50% of the overall attack rate in COVID-19 outbreaks. Furthermore, such silent transmission alone can sustain outbreaks even if all symptomatic cases are immediately isolated. The results corroborate recent contact tracing studies indicating a substantial role of presymptomatic transmission among 243 COVID-19 cases in Singapore (7) and 468 COVID-19 cases in China (8).

Our findings highlight the urgent need to scale up testing of suspected cases without symptoms as noted in revised guidelines by Centers for Disease Control and Prevention (9). Furthermore, symptom-based surveillance must be supplemented by rapid contact-based surveillance that can identify exposed individuals prior to their infectious period (10). Specifically, our estimation for isolation of silently infected individuals is a lower bound, as inevitable imperfections in isolation of symptomatic cases translates to a greater need to prevent silent transmission. Delays in contact tracing increase the risk of onward transmission, especially since those without symptoms are generally unaware of their infection risk to others, and therefore are less likely to curtail social interactions. Therefore, our estimates of the realized transmission from a silently infected individual, and their relative contribution to transmission under status quo, is likely to be conservative. These dangers are particularly salient in the context of deliberations about lifting social distancing restrictions.

Complicating future surveillance and control efforts of COVID-19 is the possibility that the seasonal drivers of influenza might comparably intensify transmission of COVID-19, such that a resurgence of COVID-19 would coincide with the next influenza season in the Northern Hemisphere. Similarities in symptoms between the two diseases may further erode the effectiveness of measures that rely on symptoms. As plans are being implemented for lifting mitigation measures, the benefits of contact-based surveillance should be evaluated to ensure adequate resources are deployed to suppress ongoing outbreaks, prevent rebound, and minimize the impact of future COVID-19 waves.

## Materials and Methods

We extended our agent-based COVID-19 transmission model (3) to include the presymptomatic phase and asymptomatic infections based on recent empirical evidence (1, 4). Each individual had an associated epidemiological status: susceptible, infected and incubating, presymptomatic, asymptomatic, symptomatic with either mild or severe illness, recovered, or dead. The daily number of contacts for each individual was sampled from an age-specific negative-binomial distribution based on an empirically determined contact matrix (11). In the absence of case isolation, each individual has 10.21 (SD: 7.65), 16.79 (SD: 11.72), 13.79 (SD: 10.50), 11.26 (SD: 9.59), and 8.00 (SD: 6.96) daily contacts in age groups 0 y to 4 y, 5 y to 19 y, 20 y to 49 y, 50 y to 64 y, and 65+ y, respectively.

Transmission was implemented probabilistically for contacts between susceptible and infectious individuals in the presymptomatic, asymptomatic, or symptomatic stages (Table 1). A proportion of infected individuals remained asymptomatic through recovery (5, 6), with an average infectious period of 5.0 d (12). The remaining proportion of infected individuals developed symptoms after an average incubation period of 5.2 d, which was sampled from a log-normal distribution (13). For symptomatic cases, the incubation period included a highly infectious presymptomatic stage prior to the onset of symptoms (1). The duration of the presymptomatic stage was sampled from a Gamma distribution with a mean of 2.3 d (1). Infectious period for symptomatic cases after the onset of symptoms was sampled from a Gamma distribution with a mean of 3.2 d (14). Among symptomatic cases, we applied an age-dependent probability of mild or severe illness (2, 3). Taking into account that infectiousness is estimated to peak 0.7 d before symptom onset (1), we calculated the transmissibility within each phase relative to the presymptomatic phase. These relative transmissibilities were estimated as 11%, 44%, and 89%, calculated using *R*_0_ components of asymptomatic, mild symptomatic, and severe symptomatic phases (4). To account for empirical uncertainty in these parameters, we sampled these values from a uniform distribution in the ranges of 0.05 to 0.16, 0.39 to 0.49, and 0.84 to 0.94, for asymptomatic, mild symptomatic, and severe symptomatic, respectively.

**Table 1.** Model parameters and their distributions

Description	Age group						Source
	0 y to 4 y	5 y to 19 y	20 y to 49 y		50 y to 64 y	≥65 y	
Transmission probability per contact during presymptomatic stage	0.0575, 0.0698	0.0575, 0.0698		0.0575, 0.0698	0.0575, 0.0698	0.0575, 0.0698	Calibrated to R_0_ = 2.5
Incubation period (days)	Log-normal (mean: 5.2, SD: 0.1)	Log-normal (mean: 5.2, SD: 0.1)		Log-normal (mean: 5.2, SD: 0.1)	Log-normal (mean: 5.2, SD: 0.1)	Log-normal (mean: 5.2, SD: 0.1)	(13)
Asymptomatic period (days)	Gamma (shape: 5, scale: 1)	Gamma (shape: 5, scale: 1)		Gamma (shape: 5, scale: 1)	Gamma (shape: 5, scale: 1)	Gamma (shape: 5, scale: 1)	Derived from ref. 12
Presymptomatic period (days)	Gamma (shape: 1.058, scale: 2.174)	Gamma (shape: 1.058, scale: 2.174)		Gamma (shape: 1.058, scale: 2.174)	Gamma (shape: 1.058, scale: 2.174)	Gamma (shape: 1.058, scale: 2.174)	Derived from ref. 1
Infectious period from onset of symptoms (days)	Gamma (shape: 2.768, scale: 1.1563)	Gamma (shape: 2.768, scale: 1.1563)		Gamma (shape: 2.768, scale: 1.1563)	Gamma (shape: 2.768, scale: 1.1563)	Gamma (shape: 2.768, scale: 1.1563)	Derived from ref. 14
Proportion of symptomatic cases with mild symptoms	0.95	0.9		0.85	0.60	0.20	(2, 3)

In the base case scenario, individuals are not isolated at any stage of infection. In order to test whether silent transmission is truly a driver of COVID-19 outbreaks, we then modeled symptom-based case isolation in which symptomatic cases were isolated immediately upon symptom onset and would remain isolated until recovery; thus, one can only transmit the disease during the presymptomatic stage. Case isolation was implemented by reducing the number of daily interactions to a maximum of three contacts, in acknowledgment that household or hospital transmission may still occur despite isolation efforts (2, 3). To identify whether outbreak control (defined as <1% cumulative incidence) could be achieved by curtailing silent transmission, we further considered isolation of presymptomatic and asymptomatic infections. We therefore simulated scenarios in which a proportion (in the range 0 to 50%) of presymptomatic and asymptomatic individuals were isolated, in addition to all symptomatic cases. The model was populated with 10,000 individuals reproducing demography for New York City. For both 17.9% and 30.8% as the asymptomatic proportion (5, 6), we calibrated the model to a reproduction number *R*_0_ = 2.5 in the absence of control measures (13). Simulations were seeded with an initial infection, and daily incidence of infection was averaged over 500 independent realizations. Model code is available at https://github.com/ABM-Lab/covid19abm.jl.

## Data Availability

The computational system and parameters are available at https://github.com/ABM-Lab/covid19abm.jl.

## References

[1] He X.., Temporal dynamics in viral shedding and transmissibility of COVID-19. Nat. Med. 26, 672–675 (2020). 32296168 10.1038/s41591-020-0869-5

[2] Moghadas S. M.., Projecting hospital utilization during the COVID-19 outbreaks in the United States. Proc. Natl. Acad. Sci. U.S.A. 117, 9122–9126 (2020). 32245814 10.1073/pnas.2004064117 PMC7183199

[3] Shoukat A.., Projecting demand for critical care beds during COVID-19 outbreaks in Canada. CMAJ 192, E489–E496 (2020). 32269020 10.1503/cmaj.200457 PMC7234264

[4] Ferretti L.., Quantifying SARS-CoV-2 transmission suggests epidemic control with digital contact tracing. Science 368, eabb6936 (2020). 32234805 10.1126/science.abb6936 PMC7164555

[5] Mizumoto K., Kagaya K., Zarebski A., Chowell G., Estimating the asymptomatic proportion of coronavirus disease 2019 (COVID-19) cases on board the Diamond Princess cruise ship, Yokohama, Japan, 2020. Euro Surveill. 25, 2000180 (2020). 10.2807/1560-7917.ES.2020.25.10.2000180 PMC7078829 32183930

[6] Nishiura H.., Estimation of the asymptomatic ratio of novel coronavirus infections (COVID-19). Int. J. Infect. Dis. 94, 154–155 (2020). 32179137 10.1016/j.ijid.2020.03.020 PMC7270890

[7] Wei W. E.., Presymptomatic transmission of SARS-CoV-2–Singapore, January 23-March 16, 2020. MMWR Morb. Mortal. Wkly. Rep. 69, 411–415 (2020). 32271722 10.15585/mmwr.mm6914e1 PMC7147908

[8] Du Z.., Serial interval of COVID-19 among publicly reported confirmed cases. Emerg. Infect. Dis. 26, 1341–1343 (2020). 32191173 10.3201/eid2606.200357 PMC7258488

[9] Centers for Disease Control and Prevention, Overview of testing for SARS-CoV-2. https://www.cdc.gov/coronavirus/2019-ncov/hcp/testing-overview.html?CDC_AA_refVal=https%3A%2F%2Fwww.cdc.gov%2Fcoronavirus%2F2019-ncov%2Fhcp%2Fclinical-criteria.html#changes, Accessed 20 June 2020.

[10] Wolfe C. M.., Ebola virus disease contact tracing activities, lessons learned and best practices during the Duport Road outbreak in Monrovia, Liberia, November 2015. PLoS Negl. Trop. Dis. 11, e0005597 (2017). 28575034 10.1371/journal.pntd.0005597 PMC5470714

[11] Mossong J.., Social contacts and mixing patterns relevant to the spread of infectious diseases. PLoS Med. 5, e74 (2008). 18366252 10.1371/journal.pmed.0050074 PMC2270306

[12] Gatto M.., Spread and dynamics of the COVID-19 epidemic in Italy: Effects of emergency containment measures. Proc. Natl. Acad. Sci. U.S.A. 117, 10484–10491 (2020). 32327608 10.1073/pnas.2004978117 PMC7229754

[13] Li Q.., Early transmission dynamics in Wuhan, China, of novel coronavirus-infected pneumonia. N. Engl. J. Med. 382, 1199–1207 (2020). 31995857 10.1056/NEJMoa2001316 PMC7121484

[14] Li R.., Substantial undocumented infection facilitates the rapid dissemination of novel coronavirus (SARS-CoV-2). Science 368, 489–493 (2020). 32179701 10.1126/science.abb3221 PMC7164387

